# The Cost-Effectiveness of Continuous Erythropoiesis Receptor Activator Once Monthly versus Epoetin Thrice Weekly for Anaemia Management in Chronic Haemodialysis Patients

**DOI:** 10.1155/2015/189404

**Published:** 2015-12-30

**Authors:** Omar Maoujoud, Samir Ahid, Hocein Dkhissi, Zouhair Oualim, Yahia Cherrah

**Affiliations:** ^1^Research Team of Pharmacoepidemiology & Pharmacoeconomics, Medical and Pharmacy School, Mohammed V University, Madinat Al Irfane, 10000 Rabat, Morocco; ^2^Department of Nephrology & Dialysis, Military Hospital Agadir, 20450 Agadir, Morocco; ^3^Meknes Dialysis Center (on Behalf of Moroccan Society of Nephrology), 33150 Meknes, Morocco; ^4^IdrissAlakbar Dialysis Center (on Behalf of the Scientific Committee, Moroccan Society of Nephrology), 12470 Rabat, Morocco

## Abstract

*Introduction*. The aim of this study was to compare the cost-effectiveness of continuous erythropoietin receptor activator (CERA) once monthly to epoetin beta (EpoB) thrice weekly to maintain haemoglobin (Hb) within the range 10.5–12 g/dL.* Methods*. Prospective cohort study and cost-effectiveness analysis. Chronic haemodialysis patients (CHP), being treated with EpoB, were selected for two periods of follow-up: period 1, maintaining prior treatment with EpoB, and period 2, conversion to CERA once monthly. Hb concentrations and costs were measured monthly. Health care payer perspective for one year was adopted.* Results*. 75 CHP completed the study, with a mean age of 52.9 ± 14.3 years. Baseline Hb was 11.14 ± 1.18 g/dL in EpoB phase and 11.46 ± 0.79 g/dL in CERA phase; we observed a significant increase in the proportion of patients successfully treated (Hb within the recommended range), 65.3% versus 70.7%, *p*: 0.008, and in the average effectiveness by 4% (0.55 versus 0.59). Average cost-effectiveness ratios were 6013.86 and 5173.64$, with an ICER CERA to EpoB at −6457.5$.* Conclusion*. Our health economic evaluation of ESA use in haemodialysis patients suggests that the use of CERA is cost-effective compared with EpoB.

## 1. Introduction

The incidence and prevalence of patients with chronic kidney disease (CKD) are growing worldwide [1, 2]. In patients with CKD, the kidneys are unable to produce enough erythropoietin to stimulate adequate production of red blood cells, causing renal anaemia [3, 4]; in addition, this anaemia is associated with reduced quality of life, high morbidity, and mortality in chronic haemodialysis patients (CHP) [5, 6]; it is commonly managed using erythropoiesis stimulating agents (ESA) [7–9]. The class of ESA includes epoetin alpha, epoetin beta (EpoB), darbepoetin, and the pegylated erythropoietin continuous erythropoiesis receptor activator (CERA) [10–12]. ESA such as epoetin alfa and EpoB require frequent administration (from three times weekly to once weekly) [13], while darbepoetin alfa can be administered once weekly or once every 2 weeks, to maintain stable Hb levels within the desired target range [14]. CERA has been recently introduced in the Moroccan market to provide correction of renal anaemia, which has unique pharmacologic properties, acting differently than short-acting EpoB at the erythropoietin receptor level [15], with a long serum half-life, allowing for once-a-month dosing. Several studies have suggested that CHP can be readily switched from short-acting ESA to CERA, but the health outcomes for patients and the effects on cost have not been extensively investigated.

The purpose of this study was to carry out a cost-effectiveness analysis (CEA) to evaluate the impact of switching patients from their current short-acting ESA therapy to CERA once monthly in a real-world setting, in this order; we conducted a multicenter prospective observational study, to compare the cost-effectiveness of CERA once monthly to treatment with EpoB thrice weekly in Moroccan haemodialysis patients.

## 2. Patients and Methods

Patients were screened at 3 haemodialysis centers in Morocco from January to December 2013; all participating centers used EpoB (Recormon; Hoffmann-La Roche Ltd., Basel, Switzerland) thrice weekly to treat their ESRD patients who had renal anaemia. To be included in the study, CHP were required to meet the following criteria: adult patients (≥18 years of age) with chronic renal anaemia, on chronic haemodialysis therapy with the same mode of dialysis for at least 12 weeks before,* Kt*/*V* ≥ 1.2, baseline Hb concentration between 10.5 and 12 g/dL; stable baseline Hb concentration, continuous subcutaneous maintenance EpoB therapy with the same dosing interval for at least 8 weeks (no change of the weekly dosage), adequate iron status defined as serum ferritin ≥200 ng/mL, or transferrin saturation ≥20%. Patients were excluded from the study when they had received an organ transplant, chemotherapy, or surgery, because they may have become anaemic for reasons other than CKD.

### 2.1. Study Design

The conversion from EpoB to CERA (methoxy polyethylene glycol-epoetin beta; Mircera; Hoffmann-La Roche Ltd., Basel, Switzerland) once monthly was already decided by the health care payer policy, who is the provider of erythropoietin stimulating agents for all patients, and was planned after a period of 6 months. Both EpoB and CERA have already been approved for renal anaemia in CHP and got the market authorization. Selected patients were not required to undergo any additional medical interventions, tests, or procedures, as they were receiving usual dialysis care and treated for renal anaemia following national and international guidelines. CHP that complied with the inclusion criteria were selected for a follow-up over two periods: the first period during six months (months −6 to 0), maintaining prior treatment with EpoB thrice weekly, and the second for six months (months 0 to 6), after changing treatment to CERA once monthly.

### 2.2. Anaemia Treatment Protocol

All enrolled patients received EpoB or CERA subcutaneously at the end of the dialysis session. The frequency of administration was 3 times a week for EpoB, and every four weeks for CERA, EpoB dosages were adjusted to maintain Hb within the recommended range 10.5–12 g/dL, at intervals of 1 to 2 weeks. Dosages were decreased by 25% for Hb increases >1 g/dL/month, versus previous level, and increased by 25% for Hb decreases >1 g/dL/month. The starting dose of CERA was based on the previous weekly dose of EpoB in the week before conversion. For patients who previously received <8000 UI of EpoB per week; the starting dose of CERA was 120 *μ*g, 200 *μ*g when previous weekly EpoB was in the range 8000–16 000 IU, and 360 *μ*g when previous weekly EpoB was >16 000 IU. Doses for all patients were to be adjusted so that haemoglobin concentrations would remain within a target range of 10.5–12 g/dL. During the follow-up, CERA dosages were decreased by 25% for Hb increases >1 g/dL/month, versus previous level, and increased by 25% for Hb decreases >1 g/dL/month, according to protocol and not more often than once monthly. Iron supplementation (iron sucrose) was to be initiated or intensified according to centre practice in cases of iron deficiency (serum ferritin <100 *μ*g/L or transferrin saturation <20%) and discontinued in patients who had serum ferritin levels >800 *μ*g/L or transferring saturation >50%.

### 2.3. Dialysis Protocol

All patients were on haemodialysis therapy using the AK 200 ULTRA-S dialysis machine (Gambro AB, Lund, Sweden). Ultrapure water was used for preparation of dialysis fluid and bicarbonate was provided from powder cartridges. Treatment time ranged from 4 h to 5 h per session, three times a week, with high-flux synthetic dialyser (UF-coefficient >20 mL/mmHg/h, surface area 1.4 to 2.1 m²). Anticoagulation was performed with low molecular weight heparin and consisted of a single dose of of 3000 to 4000 units of enoxaparin. The ultrafiltration rate was programmed to reach the patient's optimal dry weight and ranged from 500 mL/h to 900 mL/h. Water treatment system consisted of double reverse osmosis, classic pretreatment (softener, activated carbon, and microfiltration), distribution loop with permanent water circulation, and direct delivery to dialysis machines.

### 2.4. Assessments

Based on Nephrology Moroccan clinical practice guidelines [16], it is assumed that CHP treated with EpoB or CERA are monitored every 4 weeks, and the following laboratory parameters were gathered at baseline and then monthly until the end of the study: Hb, white blood cell (WBC) count, red blood cell (RBC) count, hematocrit (Hct), and platelet count, iron storage status: serum iron, and transferrin saturation (TSAT), ferritin, and biochemical profile: serum intact parathyroid hormone (iPTH), C-reactive protein (CRP), protein, albumin, total cholesterol, triglyceride, uric acid, high-density lipoprotein (HDL), low-density lipoprotein (LDL), glucose, blood urea nitrogen (BUN), creatinine (Cr), sodium (Na), potassium (K), calcium (Ca), and phosphate (P).

### 2.5. Cost-Effectiveness Analysis

The cost-effectiveness analysis (CEA) was conducted from the healthcare payer perspective, and we applied decision analytic techniques to evaluate the average and incremental cost-effectiveness of EpoB and CERA in the treatment of anaemia. Key model inputs included clinically relevant effectiveness measures, which was measured by the clinical success rate of treatment (CSR), defined as the proportion of patients successfully achieving the Hb target as well as drug acquisition costs for both treatments considered. Model outputs were expected cost-effectiveness ratio and the incremental cost-effectiveness ratio (ICER) that represents the additional cost and effectiveness obtained, when CERA regime is compared to the EpoB regime. In this order, we considered the effectiveness and cost in terms of Hb level achieved during the two periods of six months of follow-up. Two Hb ranges were considered: 10,5 to 12 g/dL (the recommended range), higher than 12 g/dL or lower than 10,5 g/dL. Measurements of Hb were performed every 4 weeks during the study period; the mean of six consecutive measures at each phase of the study was used to categorize patients on the two groups considering Hb ranges. In our analysis, the CSR was calculated at two time periods: (1) from month −6 to month 0 for EpoB and (2) from month 0 to month 6 for CERA. Costs were calculated for each patient in the 2 periods of the study, based on 24-week drug acquisition costs, and these patient-specific costs were averaged across patients within the same range of Hb.

The cost-effectiveness ratio was expressed as the mean 1-year drug costs per one per cent of EPoB or CERA patients successfully treated during the defined time period:

(1) Average  cost-effectiveness  ratio  of  treatment=Average  Cost  of  treatmentAverage  Effectiveness  treatment.

The average cost-effectiveness ratio was then compared between EpoB and CERA.

The ICER was defined as the difference in mean 1-year cost between EpoB and CERA divided by the difference in average effectiveness between the two treatments:

(2) ICER  Cera  versus  EpoB=COST  Cera−COST  EpoBEFFECTIVENESS  Cera−EFFECTIVENESS  EpoB.

### 2.6. Perspective, Timeframe, and Source of Cost Data

A health care payer perspective was adopted, for a time horizon of one year. We considered real market costs approved by the Moroccan Agency on Medical Insurance (ANAM). All costs were collected every 3 months, reported in Moroccan dirhams (MAD), then converted to US Dollar ($) (1 US Dollar = 9,297 MAD), and were inflated to 2013 costs using the consumer price index for health care goods in Morocco. A discount rate of 3% was applied to both costs and utilities. All analyses were performed using TreeAge Pro 2015 (TreeAge Software, Williamstown, MA).

### 2.7. Assumptions

It was assumed that there is no change in hospitalization attributable to both treatments, that patients make no extra doctor visits due to CERA or EpoB, as they visit the hospital for dialysis irrespective of the treatment regimen for anaemia, and that surveillance costs are assumed to be the same for both treatments.

### 2.8. Sensitivity Analysis and Monte Carlo Simulation

One-way and two-way sensitivity analyses were performed by varying baseline estimates for costs, effectiveness within a range of potentially reasonable values, and evaluating whether these changes modify the conclusions reached. Probabilistic sensitivity analysis was performed using Monte Carlo simulation (MCS) to explore overall uncertainty in the model, by creating 50,000 samples, for which expected values were calculated. Normal distributions were used for relative and baseline risks. Log-normal distributions were applied to the costing estimates.

### 2.9. Statistical Analysis

Results are expressed as percentages for discrete variables, medians, and interquartile ranges for nonnormally distributed continuous variables, and mean ± standard deviation for normally distributed continuous variables. All statistical analyses were performed using the statistical package for social sciences (SPSS) software 17.0 (SPSS, Chicago, IL, USA), and comparisons of groups were performed by Mann-Whitney or Wilcoxon test depending on the variable distributions. The study was powered to detect an incremental cost-effectiveness ratio between CERA and EpoB of less than a nominated critical threshold of three times 2013 Moroccan* per capita *gross domestic product. As almost all developing countries, there is no incremental cost-effectiveness threshold in Morocco, being considered as ideal for the acceptance of a given health intervention. So, we used the value established by the World Health Organization's Commission on Macroeconomics and Health, corresponding to three times the per capita gross domestic product (GDP), as a threshold for cost-effectiveness. According to the World Bank, the 2013 per capita GDP value was 3092$. For this reason, if a health procedure presents an ICER lower than 9186$ in Morocco, it may be considered as being cost-effective. Therefore, it was calculated that a total sample size of 70 would be sufficient to detect a 15% decrease in average cost-effectiveness ratio associated with CERA in comparison to EpoB with 80% power at *p*: 0.05.

## 3. Results

### 3.1. Patient Cohort Characteristics

**Figure 1:**
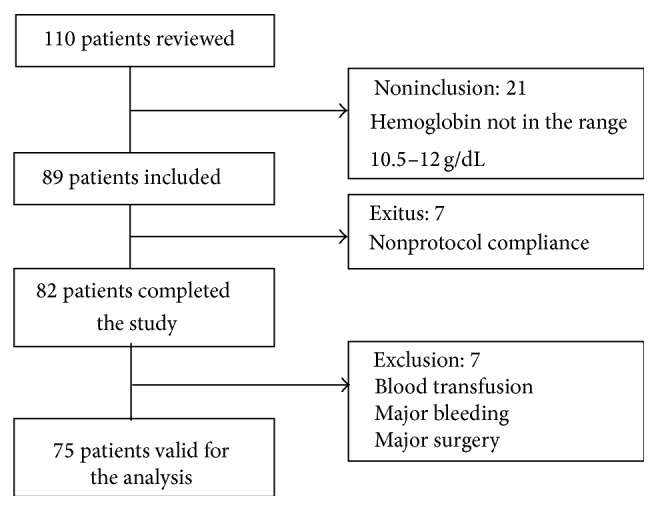
Patients included in the study.

We screened a total of 110 patients; 89 of them complied with the inclusion criteria (Figure 1); screen failures were due to an Hb not in the recommended range. In total, 82 patients have completed the study and 75 were valid for the analysis (48 (64.9%) men and 27 (35.1%) women), with a mean age of a mean age of 52.9 ± 14.3 years; the most common reasons for exclusion were active bleeding (major trauma, gastric ulcer bleeding, or surgery).  Table 1 summarizes demographic and baseline characteristics of valid patients, diabetic nephropathy was the most common cause of renal disease (28.2%), and 65.4% of the patients had a history of cardiovascular disease. Vascular access was an arteriovenous fistula in the majority of patients 70 (93%), and 5 patients were treated via a permanent catheter.

### 3.2. Efficacy Evaluation

**Figure 2:**
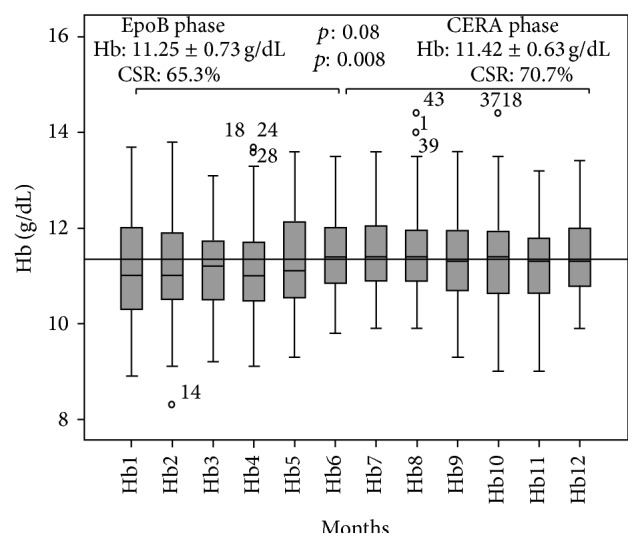
Evolution of mean Hb concentration during the two phases of the study. Hb: hemoglobin, EpoB recombinant human erythropoietin beta, CERA: continuous erythropoietin receptor activator, and CSR: clinical success rate (HB within the target 10,5–12 g/dL).

Baseline Hb level was 11.14 ± 1.18 g/dL in EpoB phase and 11.46 ± 0.79 g/dL in CERA phase; there was a nonsignificant increase in mean Hb level after conversion to CERA (11.25 ± 0.73 versus 11.42 ± 0.63 g/dL *p*: 0.08), but we observed a significant increase in the proportion of patients successfully treated (CSR 65.3% versus 70.7% *p*: 0.008). Furthermore, the proportion of patients with Hb < 10.5 g/dL was 14% in EpoB phase and 7% in CERA phase, and the proportion of patients with Hb > 12 g/dL was 12% and 15%, respectively, without need of blood transfusions during the two periods of follow-up. The average effectiveness rose by 4% (0.55 versus 0.59). Monthly evolution of Hb during the two phases of the study is reported in Figure 2.

### 3.3. EPO and Iron Requirements

The mean weekly dose during the EpoB period was 6104 ± 3178 ui and was 106.4 ± 50.1 *μ*g/month in the CERA phase; there was no significant difference in the proportions of patients receiving IV iron during the two periods of follow-up (87.5% and 89.1%, resp., *p*: 0.23), and conversion from EpoB to CERA did not result in statistically significant change in mean serum ferritin, TSAT, or iron dose as reported in Table 2. There were no significant differences between EpoB and CERA periods in terms of dialysis doses (*Kt*/*v *1.26 ± 0.4 versus 1.27 ± 0.1, *p*: 0.1) and duration of HD sessions (244.5 ± 12.4 versus 243.5 ± 11.4 mn, *p*: 0.34), also there were no significant changes in inflammatory parameters: serum C-reactive protein (3.3 ± 1.1 versus 3.7 ± 0.9 mg/L, *p*: 0.11) and albumin levels (3.8 ± 1.8 versus 4.1 ± 0.7 g/L, *p*: 0.21). Patients with permanent central venous catheter had a nonsignificant higher dose of ESA in comparison to patient with arteriovenous fistula (6107.5 ui versus 6101.2 ui, *p*: 0.1) for EpoB and (110.4 versus 102.5, *p*: 0.2) for CERA.

### 3.4. Cost Effectiveness Analysis

**Figure 3:**
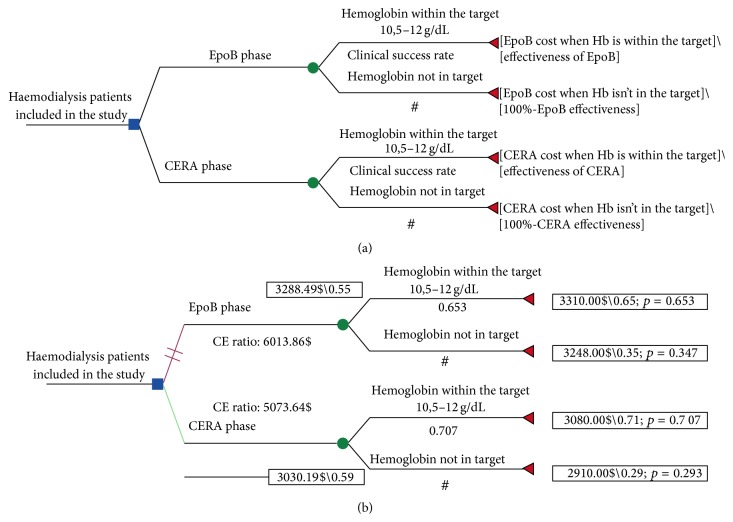
(a) Decision tree framework. (b) Results of cost-effectiveness analysis after roll-back calculation. EpoB: recombinant human erythropoietin beta, CERA: continuous erythropoietin receptor activator, CE ration: cost-effectiveness ratio.

**Figure 4:**
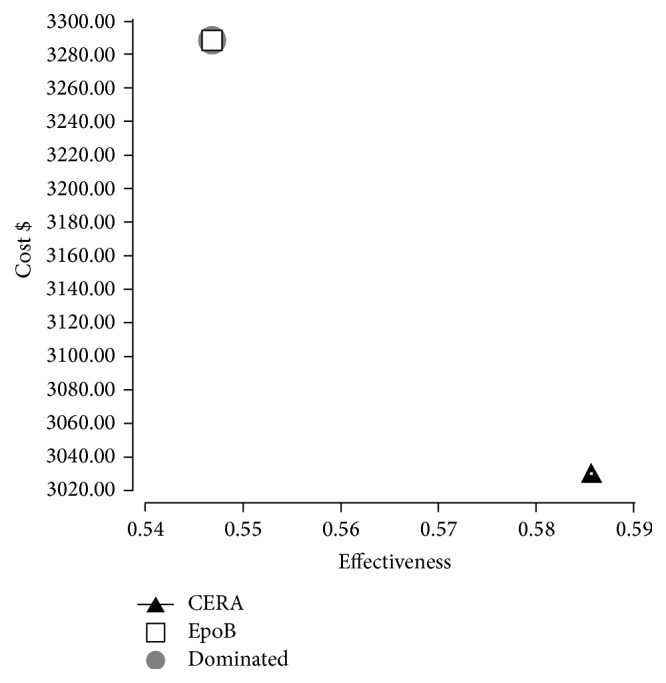
Cost-effectiveness diagram. EpoB is dominated by CERA since it is more costly and less effective.

Decision tree framework is presented in Figure 3(a), with CERA and EpoB branches, and the rolled back model with calculations is presented in Figure 3(b). Costs, effectiveness, and incremental associated with CERA administration to CHP, compared to EpoB, are summarized in Table 3. Based on 6-month drug acquisition cost, the mean per patient cost was 1644.2 ± 859.4$ for EpoB and 1515.5 ± 713$ for CERA, projected annual per patient costs were 3288.49 ± 1718.9$ and 3030.19 ± 1426$, respectively, with a cost saving associated with CERA at −258.3$. Average annual effectiveness estimated by the model was 0.55 for EpoB and 0.59 for CERA, and average cost-effectiveness ratios were 6013.86$ and 5173.64$, respectively, with an ICER at −6457.5$ per one per cent of patients successfully treated, so the treatment with EpoB was dominated by CERA as shown in the cost-effectiveness diagram Figure 4.

### 3.5. Sensitivity Analyses and Monte Carlo Simulation

**Figure 5:**
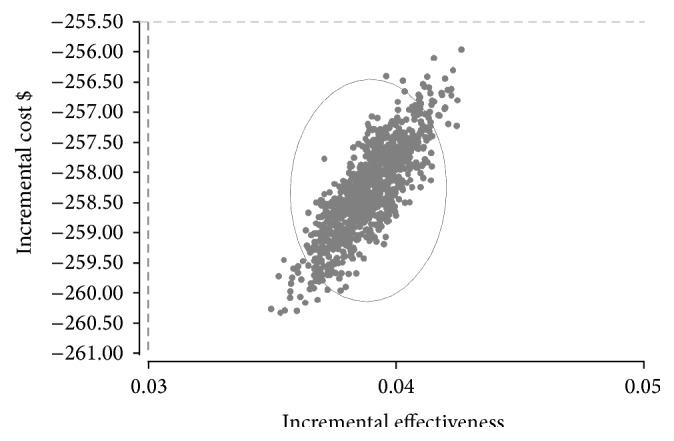
Results of Monte Carlo simulation of incremental cost and effectiveness scatter plot of CERA versus EpoB, based on 50000 random iterations of cost-effectiveness model.

**Figure 6:**
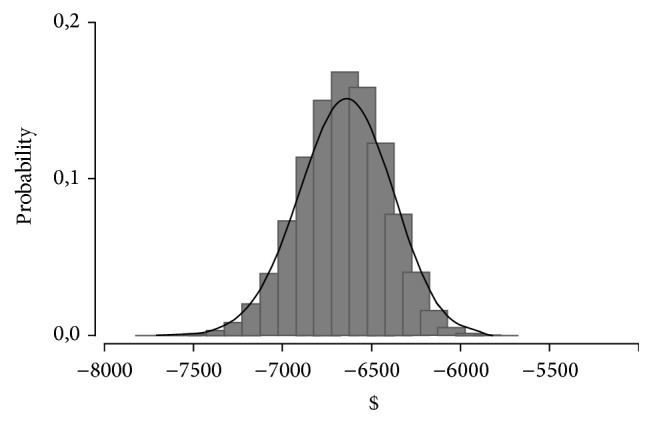
Probabilistic sensitivity analysis of ICER distribution of CERA versus EpoB. The analysis confirmed the robustness of the model, based on 50 000 random iteration; the ICER remain within the range of calculated ICER ± 10%.

We performed one-way and multiway sensitivity analysis, the ICERs did not change significantly, despite application of variation rates of ±10% in each of the parameters of cost and effectiveness individually, and clinically implausible changes in variables were required to significantly improve the cost effectiveness EpoB in comparison to CERA. The results of probabilistic sensitivity analysis by Monte Carlo simulation based on 50000 random iterations are shown in Figure 5. The scatterplot illustrates the robustness of the model. Analysis of the results shows that 100% of the points are in the lower right hand portion of the graph (best effectiveness and lower cost), demonstrating that CERA remained more effective, less costly, and hence the dominant treatment compared to EpoB. Also, the robustness of the calculated ICER was confirmed by MCS as shown in Figure 6.

## 4. Discussion

The present study found that CERA is more cost-effective than EpoB, making it the dominant treatment for the management of anaemia in chronic haemodialysis patients. To our knowledge, this work is the first to evaluate the cost-effectiveness of CERA prospectively in a real-life practice. As demonstrated in the recent review published by Schmid [14], the literature query of studies dealing with the cost or the cost-effectiveness of CERA was poor; only 18 publications were included in his analysis, most of the available data was from meeting abstracts (eleven), and only seven published studies were in peer-reviewed journals. Majority of included studies were retrospectives, and reported data were only about cost of therapy after a switch to CERA from single-center experiences. Gonzalez et al. [17] reported in a meeting abstract a CEA of CERA compared to erythropoietin alpha, on the base of a decision tree model that simulated the treatment costs and outcomes in Mexican haemodialysis patients. In this study, the clinical success rate (patients within 11–12.5 Hb/dL levels) when using CERA versus EPO-alpha showed significant difference (86.79% versus 50.48% resp., *p* < 0.0001), treatment care cost per year for CERA was $2,776.13 versus $2,907.88 for erythropoietin alpha (*p* < 0.0001), and the cost-effectiveness plane indicates that CERA is a highly cost-effective therapy, with a probability of 0.60 to be cost saving and 0.99 of probability of being cost effective. In another study published as an abstract, aimed to determine the cost-effectiveness of anaemia treatment in dialysis patients for Brazilian Public Health System [18], using a Markov model of a hypothetical cohort of dialysis patients treated with CERA or epoetin for four years, the model showed that epoetin treatment was more cost-effective than CERA treatment. Unfortunately, it was not possible to evaluate the methodology of both previous publications and there concordance with international guidelines for CEA studies [19]. Considering only the cost of treatment; 3 cost-minimisation studies reported as meeting abstracts confirm our finding of cost saving after switch to CERA from another short acting EPO; Bezditko et al. [20] estimated the cost reduction about 5–35%, based on decision tree analysis. In his pharmacoeconomic evaluation of maintenance treatment of anaemia, in Ukrainians haemodialysis patients, the average costs of CERA treatment per patient on haemodialysis were $173/week (intravenous route of administration) and $130/week (subcutaneous route of administration) and average costs for using the shorter-acting EpoB drugs were $267–194/week and $133–182/week, respectively. Franz et al. [21], in a Swiss multicenter prospective observational study, analysed data of dialysis patients treated with ESA over a period of 12 months. After the switch to CERA from treatment with either darbepoetin alfa or epoetin alfa/beta, the cost of ESA treatment decreased by 14% and patients maintained stable Hb values in the first 6 months after conversion. In contrast, Albero Molina et al. [22] reported a +66.4% increasing cost after switch to CERA, in a 6-month prospective follow-up of 17 haemodialysis patients, with stable dose of subcutaneous EpoB average costs/patient/month: EpoB (*€*174.30 ± *€*85.40) versus CERA (*€*290.10 ± *€*69.00). In another Spanish study, Escudero-Vilaplana et al. [23] reported similar cost increase after switch to CERA from EpoB €103.2 versus €147.5. In our analysis, the cost reduction related to CERA can partially be explained by the lower doses required after conversion from EpoB. Initial dose was calculated according to manufacturer guidelines, and during the follow-up doses adjustments were permitted according to Hb evolution. We believe that this work is the first to report data related to CERA use in such North African ethnic population, similarly to our finding in another Mediterranean population, authors reported low dose requirement of CERA (mean monthly dose was 112.4 ± 76.78 *μ*g) to maintain Hb in the range 10–12 g/dL [24]. Also, low dose requirement in CERA phase can be explained by an increase in iron use in terms of proportion of patients receiving iron, serum ferritin, TSAT, and mean iron doses, but this improvement did not reach statistical significance in comparison to EpoB period. Of note, majority of available data suggests cost saving after conversion to CERA, rather than cost increase; it was difficult to have a conclusion from these previous studies. Since they were surrounded by considerable uncertainty and unavailable full-text articles, few abstracts reported information about baseline and evolution of Hb, ESA doses, median cost/patient, and iron status, and majority of analyses were based on hypothetical cohorts rather than real-life follow-up. For this reasons, we have tried in our analysis to follow the guidelines of the International Society for Pharmacoeconomics and Outcomes Research (ISPOR), health economic evaluation publication guidelines, and Consolidated Health Economic Evaluation Reporting Standards (CHEERS) [19]. Results from a number of previous studies suggested that adoption of a once-monthly ESA could provide considerable time savings for dialysis centers [25–27], in our study; we did not consider healthcare personnel time associated with routine anaemia management tasks, since we adopted the health care payer perspective. However, our study has some limitations. First, the perspective was that of a health-care payer and not a societal one, and as such we did not include indirect costs such as loss of productivity and travel costs. The absence of evidence that ESA use increases employment rats in haemodialysis patients makes it unlikely that adopting societal perspective would have changed our results [5, 28, 29]. Secondly, we considered only drugs acquisition costs, without including other direct medical costs such hospitalization, medications, and consultations; however, previous research indicated that these additional cost components would be similar between CERA and EpoB [30]. In our CEA, we considered the effectiveness in terms of Hb target reached, rather than a hard end point like mortality or change in quality of life (QOL), the initial design of the study was not appropriate to evaluate the cost per quality adjusted life year (QALY), and the short duration of follow-up was not adequate to expect a significant change in QOL or in mortality related to conversion to CERA. For this reason, we adopted a valid surrogate marker such as the CSR, since a strong correlation between QOL and Hb levels in CHP is admitted now.

In summary, this medicoeconomic evaluation of ESA use in haemodialysis patients suggests that administering CERA once monthly is cost-effective when the health care perspective is employed. We performed our study in accordance with current anaemia management guidelines in haemodialysis patients, and following Health Economic Evaluation Reporting Standards. These data would assist health care decision makers and services reimbursement authorities as they aim to provide the most cost-effective treatments to patients.

## Figures and Tables

**Table 1:** Baseline demographic and clinical characteristics of the haemodialysis population.

Parameters	
Patients (*n*)	75
Age (years)	56.6 ± 11.77
Male (%)	48 (64.9%)
Primary renal disease *n* (%)	
Diabetes	22 (28.2)
Glomerulonephritis	10 (12.8)
Unknown	24 (30.7)
Vascular	12 (15.3)
Others	10 (12.8)
Vascular access *n* (%)	
Fisulta	70 (93)
Catheter	5 (7)
Viral hepatitis B or C *n* (%)	0
Patients on antihypertensive therapy *n* (%)	35 (46.66)
Statins *n* (%)	31 (41.33)
Time on dialysis (months)	75.2 ± 25.6
Predialysis systolic BP (mmHg)	138 ± 21.6
Predialysis diastolic BP (mmHg)	81 ± 2,6
Dry weight (kg)	68.2 ± 7.6
Haemoglobin (g/dL)	10.91 ± 1.56
Albumin (g/L)	3.9 ± 0.82
C-reactive protein (mg/L)	5.25 [1.9–11.6]
Ferritin (ng/mL)	392.64 ± 250
Transferrin saturation (%)	29.5 ± 5.4
*Kt*/*V*	1.25 [1.06–1.57]
Dialysis session length	243.7 ± 15.6
Intact parathyroid hormone (pg/mL)	362.7 [170.9–521.2]
Calcium (mg/dL)	9.39 ± 0.71
Phosphate (mg/dL)	4.33 ± 1.83
Cholesterol (mg/dL)	166.2 ± 49.2
LDL	94.34 ± 33.2
HDL	40.79 ± 20.6
Triglyceride (mg/dL)	130.5 ± 23.6

**Table 2:** Anaemia management during the study.

Parameters	EpoB phase	CERA phase	*p*
*n*	75	75	

Hemoglobin g/dL	11.25 ± 0.73	11.42 ± 0.63	0.08

Transferrin saturation %	28.4	29.2	0.35

Ferritin ng/mL	288.5	299.3	0.2

IV iron %	87.5	89.1	0.23
IV iron dosage mg/month	110,5 ± 11.2	116,3 ± 13.3	0.11

ESA dose	6104 ± 3178 ui	106.4 ± 50.1 *μ*g	

CSR *n* %	49 (65.3)	53 (70.7)	0.008

Hb > 12 g/dL *n* (%)	12 (16)	15 (20)	0.001

Hb < 10.5 g/dL *n* (%)	14 (18.7)	7 (9.3)	0.001

CSR: clinical success rate Hb within the range 10.5–12 g/dL.

ESA: erythropoietin stimulating agent.

Hb: haemoglobin.

EpoB: epoetin beta.

CERA: continuous erythropoietin receptor activator.

**Table 3:** Cost-effectiveness analysis of the study.

Parameters	EpoB	CERA	*p*
*N*	75	75	
Patients successfully treated *n* (%) Hb 10.5–12 g/dL	49 (65.3)	53 (70.7)	0.008
Patients not successfully treated *n* (%) Hb > 12 or Hb < 10.5 g/dL	26 (34.7)	22 (29.3)	0.001
Drug costs $			
Mean 6-month drug cost per patient	1644.2 ± 859.4	1515.5 ± 713	0.03
Mean 1-year drug cost per patient	3288.49	3030.19	0.03
Incremental 1-year cost Cera versus EpoB $	—	−258.3	
Average effectiveness	0.55	0.59	
Incremental effectiveness Cera versus EpoB $	—	+0.04	
Average cost-effectiveness ratio $/per one per cent of patients successfully treated	6013.86	5173.64	
ICER Cera versus EpoB $/per one per cent of patients successfully treated		−6457.5	

ESA: erythropoietin stimulating agent.

Hb: haemoglobin.

EpoB: epoetin beta.

CERA: continuous erythropoietin receptor activator.

ICER: incremental cost-effectiveness ratio.
